# Long-lived Snell dwarf mice display increased proteostatic mechanisms that are not dependent on decreased mTORC1 activity

**DOI:** 10.1111/acel.12329

**Published:** 2015-02-26

**Authors:** Joshua C Drake, Danielle R Bruns, Frederick F Peelor, Laurie M Biela, Richard A Miller, Benjamin F Miller, Karyn L Hamilton

**Affiliations:** 1 Health and Exercise Science Department, Colorado State University220 Moby B Complex, Fort Collins, CO, 80523-1582, USA; 2 Department of Pathology and Geriatrics Center, University of Michigan109 Zina Pitcher Place, Ann Arbor, MI, 48109-2200, USA

**Keywords:** DNA synthesis, long-lived model, protein synthesis, proteostasis, stable isotope

## Abstract

Maintaining proteostasis is thought to be a key factor in slowed aging. In several growth-restricted models of long-life, we have shown evidence of increased proteostatic mechanisms, suggesting that proteostasis may be a shared characteristic of slowed aging. The Snell dwarf mouse is generated through the mutation of the *Pit-1* locus causing reductions in multiple hormonal growth factors and mTORC1 signaling. Snell dwarfs are one of the longest lived rodent models of slowed aging. We hypothesized that proteostatic mechanisms would be increased in Snell compared to control (Con) as in other models of slowed aging. Using D_2_O, we simultaneously assessed protein synthesis in multiple subcellular fractions along with DNA synthesis in skeletal muscle, heart, and liver over 2 weeks in both sexes. We also assessed mTORC1-substrate phosphorylation. Skeletal muscle protein synthesis was decreased in all protein fractions of Snell compared to Con, varied by fraction in heart, and was not different between groups in liver. DNA synthesis was lower in Snell skeletal muscle and heart but not in liver when compared to Con. The new protein to new DNA synthesis ratio was increased threefold in Snell skeletal muscle and heart compared to Con. Snell mTORC1-substrate phosphorylation was decreased only in heart and liver. No effect of sex was seen in this study. Together with our previous investigations in long-lived models, we provide evidence further supporting proteostasis as a shared characteristic of slowed aging and show that increased proteostatic mechanisms may not necessarily require a decrease in mTORC1.

## Introduction

Protein turnover decreases with age, resulting in a progressive accumulation of damaged proteins and propagation of the aging phenotype (Stadtman, 1988, 1992). Maintaining protein homeostasis (i.e., proteostasis) through coordination of mRNA translation, protein synthesis, protein folding, and protein breakdown may be a key component of slowed, or healthy aging (Orgel, 1963; Treaster *et al*., 2013; Miller *et al*., 2014). Therefore, models of slowed aging may provide valuable insight into the role of proteostasis and how proteostatic mechanisms are regulated during slowed aging.

As enzymatic capacity for protein repair is limited, cells must use alternative means for dealing with the age-dependent accumulation in protein damage (Mortimore & Poso, 1987; Mary *et al*., 2004). In tissues where apoptosis is maladaptive in dealing with accumulated damage due to a limited ability to create new cells (e.g., skeletal muscle), damaged proteins must be removed through autophagy and/or proteolysis and replaced through the synthesis of new proteins (Mortimore & Poso, 1987; Mary *et al*., 2004; Poppek & Grune, 2006). Thus, the synthesis of new proteins is essential for maintaining cellular proteostasis (Treaster *et al*., 2013). Although protein synthesis is a critical component of proteostasis, measuring protein synthesis alone is not sufficient to determine changes in proteostatic mechanisms (Miller *et al*., 2014). For example, during cell division, increases in protein synthesis are required to ensure an equal complement of proteins between daughter cells and serve to essentially ‘dilute’ protein damage (Eden *et al*., 2011). In contrast, adaptation to extra- and intracellular environmental stimuli requires increases in protein synthesis to maintain cellular homeostasis but does not necessarily include cell replication. We have proposed that simultaneously assessing both protein and DNA synthesis through deuterium oxide incorporation (D_2_O) can provide insight into what proportion of new proteins is made in new versus existing cells (Drake *et al*., 2014; Miller *et al*., 2014).

The mTOR signaling pathway integrates nutrient and hormonal signaling to regulate protein turnover (e.g., protein synthesis, cell cycle, and autophagy) via two multiprotein complexes, mTORC1 and mTORC2 (Laplante & Sabatini, 2012). Lifelong caloric restriction (CR) is associated with decreased mTORC1 signaling, while chronic administration of rapamycin (Rap), an mTORC1 inhibitor, increases the lifespan (Turturro *et al*., 1999; Harrison *et al*., 2009). Using D_2_O in both long-lived CR and Rap models, we found evidence of increased proteostatic mechanisms, demonstrated as an increase in the new protein to new DNA synthesis ratio (Miller *et al*., 2014), alongside decreased mTORC1 signaling (Miller *et al*., 2012a,b; Drake *et al*., 2013). More recently in the long-lived crowded litter mouse model, we showed a similar increase in proteostatic mechanisms despite an increase in mTORC1 signaling (Drake *et al*., 2014). These data suggest that decreased mTORC1 signaling may not be necessary for increased proteostatic mechanisms in models of long-life.

The growth-restricted Snell dwarf mouse model has a mutation of the *Pit-1* locus that results in an underdeveloped anterior pituitary, causing reductions in anterior pituitary hormone production (e.g., growth hormone, thyroid stimulating hormone, and prolactin) and inhibition of upstream regulators of mTORC1 and phosphorylation of downstream substrates in some tissues (Hsieh *et al*., 2002; Hsieh & Papaconstantinou, 2004). Compared to their respective controls, the Snell dwarf (as well as the analogous Ames dwarf) mouse has the greatest increase in lifespan among mouse models of slowed aging (Brown-Borg *et al*., 1996), with female Snell dwarfs significantly outliving their male counterparts. We hypothesized that proteostatic mechanisms, as assessed by the ratio of new protein to new DNA synthesis, would be greater in Snell compared to normal controls (Con) in multiple tissues and would correspond with a decrease in mTORC1-related signaling in the Snell mice. We also assessed these parameters by sex to determine whether proteostasis differed between sexes.

## Results

### Protein and DNA synthesis

**Fig 1:**
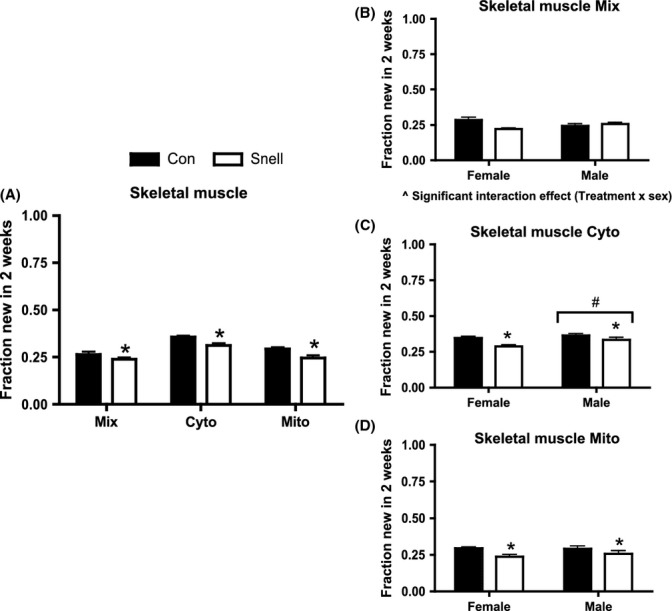
Protein synthesis in skeletal muscle and between sexes over 2 weeks in Snell and Con. Protein synthesis was significantly decreased in Snell compared to Con in Mix, Cyto, and Mito fractions (A). In Mix, there was a significant interaction between treatment (Snell) and sex (B). Protein synthesis was decreased in both female and male Snell compared to their respective Con in Cyto and Mito (C, D). Independent of treatment, Cyto protein synthesis was increased in males compared to females (C). *n* = 5 per sex and *n* = 10 per group. *P < 0.05 for Snell vs. Con; #*P* < 0.05 difference between sexes independent of treatment; and ^*P* < 0.05 Interaction between treatment and sex.

In skeletal muscle (gastroc complex - whole gastrocnemius, soleus, and plantaris homogenate), Snell dwarf mice had significantly lower rates of protein synthesis in Mix, Cyto, and Mito compared to Con (Fig.1A). In the Mix fraction (plasma membrane, nuclei, and contractile proteins) there was a significant interaction between genotype (Snell versus Con) and sex (Fig.1B); the decline in protein synthesis in the Snell mice was seen in females only. There were no interactions with sex in protein synthesis in Cyto (cytosolic organelles with the exception of mitochondria) or Mito (mitochondria enriched) fractions (Fig.1C,D). Independent of treatment, males had significantly greater rates of protein synthesis in Cyto compared to females (Fig.1C).

**Fig 2:**
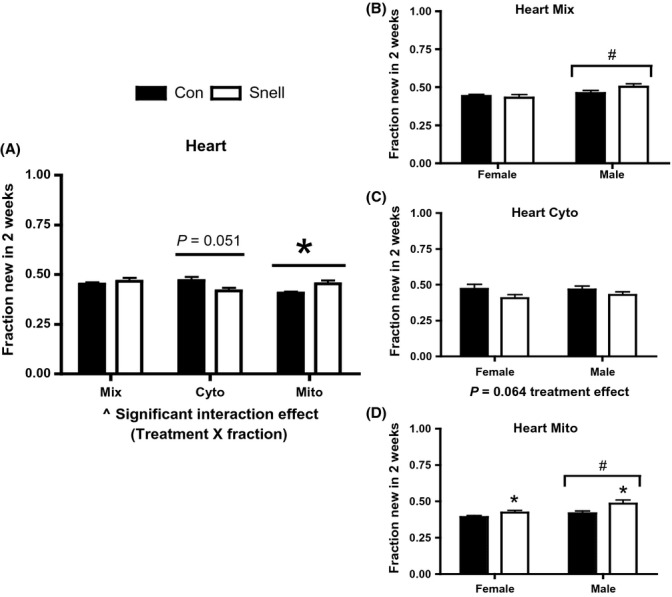
Protein synthesis in heart and between sexes over 2 weeks in Snell and Con. There was a significant interaction in protein synthesis between treatment (Snell) and fraction (A). Mix protein synthesis was significantly increased in pooled males compared to pooled females (B). A trend (*P* = 0.064) of decreased protein synthesis was observed for both sexes in Cyto (C). Mito protein synthesis was increased in both sexes (C). Males independent of treatment had increased protein synthesis compared to females (C). *n* = 5 per sex and *n* = 10 per group. *P < 0.05 for Snell vs. Con; #*P* < 0.05 difference between sexes independent of treatment; and ^*P* < 0.05 Interaction between treatment and fraction.

In heart, there was a significant interaction between treatment (Snell versus Con) and subcellular fraction (Fig.2A). Mix protein synthesis in heart was not different between groups (Fig.2A). Rates of Mito protein synthesis in heart were significantly greater in Snell compared to Con (Fig.2A). Cyto protein synthesis in heart was less in Snell compared to Con, but this did not reach statistical significance (*P* = 0.051) (Fig.2A). When separated by sex, there was not a significant difference between Con and Snell in heart Mix or Cyto fractions (Fig.2B,C). In both female and male Snell, heart Mito protein synthesis was greater compared to their respective Con (Fig.2D). Independent of treatment in the heart, males had significantly greater rates of protein synthesis in the Mix and Mito fractions compared to females (Fig.2B,D).

**Fig 3:**
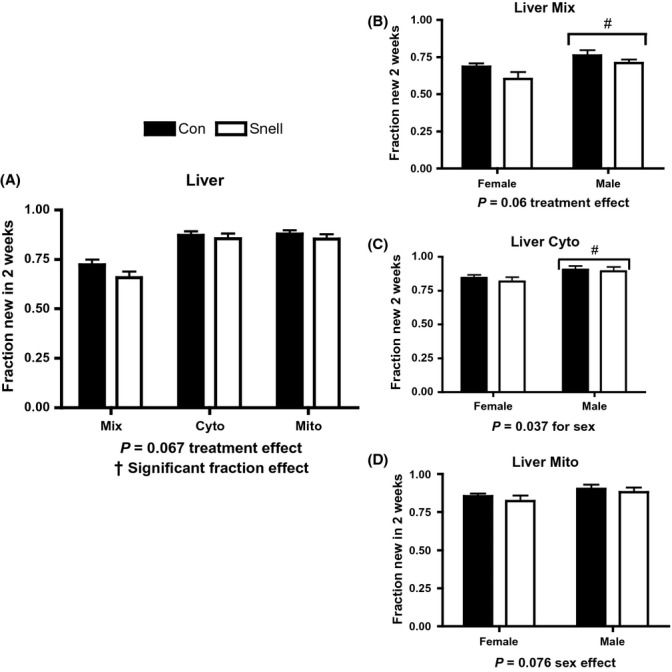
Protein synthesis in liver and between sexes over 2 weeks in Snell and Con. A trend (*P* = 0.067) of decreased protein synthesis was observed across fractions in Snell compared to Con, and protein synthesis was significantly different between fractions (A). A trend (*P* = 0.060) for decreasing protein synthesis in both sexes within Mix was observed; also, Mix protein synthesis was significantly higher in males compared to females, independent of treatment (B). Males had higher Cyto protein synthesis compared to females, independent of treatment (C). In Mito, there was a trend (*P* = 0.076) for increased protein synthesis in males compared to females independent of treatment (D). *n* = 5 per sex and *n* = 10 per group, except for Con Cyto *n* = 9 corresponding to *n* = 4 in male Con Cyto. *P < 0.05 for Snell vs. Con; †*P* < 0.05 difference between fractions; and #*P* < 0.05 difference between sexes independent of treatment.

In liver, Snell had a trend (*P* = 0.067) for lower protein synthesis in all subcellular fractions assessed compared to Con (Fig.3A). When separated by sex, there was a trend (*P* = 0.060) for Snell having lower rates of protein synthesis in the Mix fraction (Fig.3B). Independent of treatment, liver, protein synthesis was significantly greater in males compared to females in Cyto and trended toward an increase in Mix (*P* = 0.06) and Mito fractions (*P* = 0.076) (Fig.3B–D).

**Fig 4:**
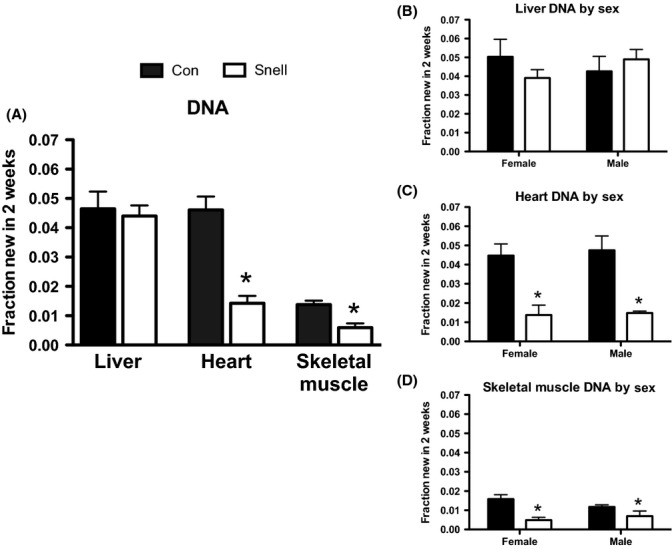
DNA synthesis in skeletal muscle, heart, and liver and between sexes over 2 weeks in Snell and Con. DNA synthesis was significantly decreased in skeletal muscle and heart, but no difference was observed in liver DNA synthesis between Snell compared to Con (A). When separated by sex, the decrease in DNA synthesis in Snell compared to Con was conserved in females and males in skeletal muscle and heart (B, C). No sex differences in DNA synthesis were observed in the liver (D). *n* = 5 per sex and *n* = 10 per group. *P < 0.05 for Snell vs. Con.

DNA synthesis rates were significantly lower in Snell compared to Con in both heart and skeletal muscle, but were not different from Con in liver (Fig.4A). Rates of DNA synthesis were also lower in Snell compared to Con when the data were separated by sex (Fig.4B–D).

**Fig 5:**
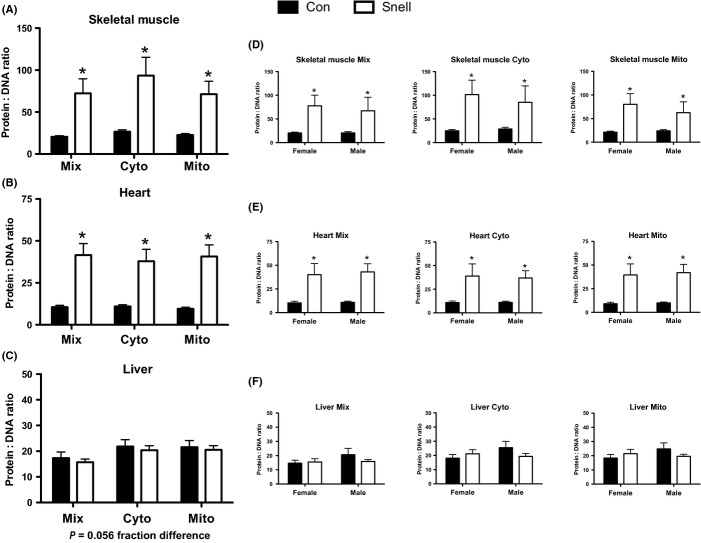
Protein to DNA synthesis ratios in skeletal muscle, heart, and liver and between sexes in Snell and Con. In skeletal muscle and heart, there was a significant increase in the protein to DNA ratio in Mix, Cyto, and Mito fractions (A, B), which was conserved in both sexes (C, D). No change in the protein to DNA ratio between Snell and Con was observed in liver or when separated by sex (E, F). *n* = 5 per sex and *n* = 10 per group. *P < 0.05 for Snell vs. Con.

In both skeletal muscle and heart, there was a significant increase in the new protein to new DNA synthesis ratio in Snell compared to Con (Fig.5A,C), which was not different between sexes (Fig.5B,D). In the liver, there was no difference in the new protein to new DNA synthesis ratio between Snell and Con or when separated by sex (Fig.5E,F).

### mTORC1 signaling

**Fig 6:**
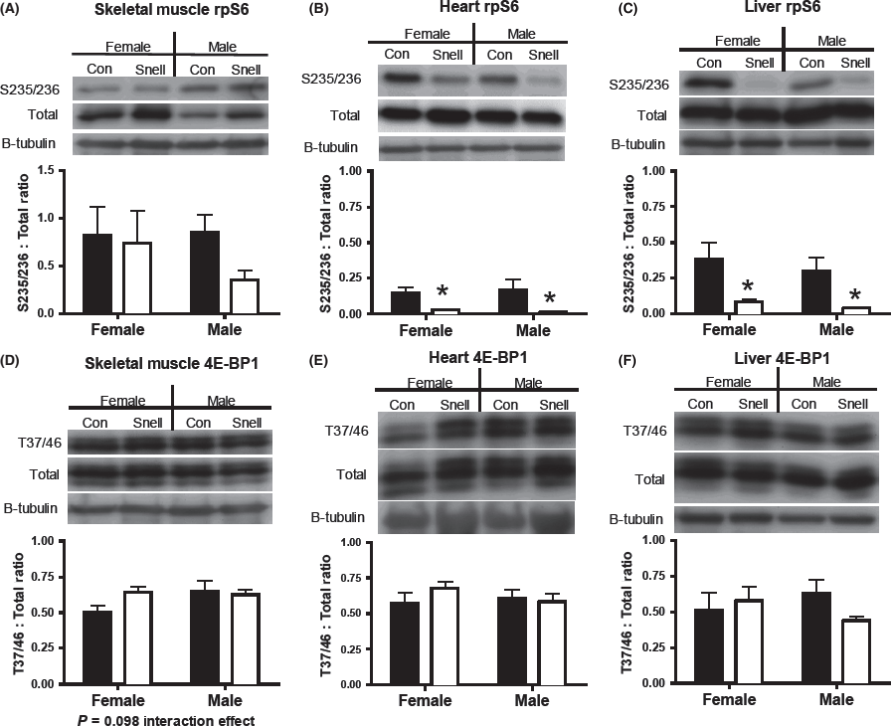
Western blotting of mTORC1 substrates rpS6 and 4E-BP1 in skeletal muscle, heart, and liver of Snell and Con. In skeletal muscle, rps6 phosphorylation was not different between Snell and Con or between sexes. rpS6 phosphorylation was significantly decreased in heart and liver, which was conserved in both sexes (B, C). There was a slight interaction trend in skeletal muscle (*P* = 0.098) 4E-BP1 between treatment (Snell) and sex (D). No changes in 4E-BP1 were found in skeletal muscle, heart, or liver between Snell and Con (E, F). *n* = 5 per sex and *n* = 10 per group. *P < 0.05 for Snell vs. Con.

Phosphorylation of the mTORC1 substrate, ribosomal protein S6 (rpS6), was significantly decreased in heart and liver of Snell compared to Con in both sexes (Fig.6B,C). In skeletal muscle, there was no difference in rpS6 phosphorylation between Snell and Con (Fig.6A). Phosphorylation of another mTORC1 substrate, eukaryotic initiation factor 4E binding protein 1 (4E-BP1), was not statistically different in any tissue or between sexes (Fig.6D–F), though a slight interaction trend between treatment (Snell vs. Con) and sex was observed in skeletal muscle (*P* = 0.098) (Fig.6D,E).

## Discussion

Here, we present a tissue- and sex-specific assessment of proteostasis using DNA and protein synthesis in long-lived Snell dwarf mice. We tested the hypothesis that Snell mice would demonstrate increases in proteostatic mechanisms across multiple tissues; consistent with our findings in other slowed aging models (Miller *et al*., 2012a,b, 2014; Drake *et al*., 2013, 2014). We demonstrate that proteostatic mechanisms, as assessed by the new protein to new DNA synthesis ratio, were increased by threefold in skeletal muscle and heart of Snell compared to normal controls, but independent of sex. Additionally, the mTORC1-substrate, rpS6, was decreased in heart and liver, but not in skeletal muscle. Collectively, our data further suggest proteostasis is a shared characteristic of slowed aging (Miller *et al*., 2012a,b; Drake *et al*., 2013, 2014) but may not necessarily be dependent on decreased mTORC1 signaling.

### Proteostasis in Snell dwarf mice

The synthesis of new proteins to replace damaged, and subsequently removed, proteins is an essential component of maintaining proteostasis (Mortimore & Poso, 1987; Mary *et al*., 2004). In this study, we used D_2_O to simultaneously assess both new protein and new DNA synthesis across multiple tissues and subcellular fractions, as well as between sexes (Neese *et al*., 2002; Busch *et al*., 2006; Robinson *et al*., 2011; Miller *et al*., 2012a,b; Drake *et al*., 2013) in the long-lived Snell dwarf mouse. We show that protein synthesis is decreased in all subcellular fractions, including Mito, in skeletal muscle. A decrease in mitochondrial protein synthesis, that is, decreased mitochondrial biogenesis (Miller & Hamilton, 2012), is counter to the idea of slowed aging (Rooyackers *et al*., 1996), although markers of mitochondrial biogenesis (e.g., NRF-1 and TFAM gene expression) have been shown to be decreased in the long-lived GHRKO mice (Gesing *et al*., 2013). However, when decreased mitochondrial protein synthesis, as well as protein synthesis in other cell fractions, is considered in relation to the reduced DNA synthesis [indicative of S-phase synthesis, not repair (Neese *et al*., 2002)] in Snell skeletal muscle, the ratio of new protein to new DNA synthesized is approximately threefold greater in Snell compared to Con. To us, this suggests that the synthesis of new proteins is occurring primarily in existing cells (Miller *et al*., 2014). Thus, new proteins may be directed toward maintaining existing cellular structures (including mitochondria), in Snell dwarf skeletal muscle, which is consistent with slowed aging.

In the heart, the protein synthetic response varies between subcellular fractions in Snell compared to Con. Again, when considered in relation to the decrease in new DNA synthesized in the heart, the new protein to new DNA synthesis ratio is increased threefold across fractions in Snell despite the differential protein synthesis response between fractions compared to Con. Our findings of increased proteostatic mechanisms in Snell mice are in accordance with long-lived CR, Rap, and CL mouse models of the same age (model and tissue comparisons are summarized in Table S1) (Miller *et al*., 2012a,b; Drake *et al*., 2013, 2014). It is worth highlighting that the increase in the new protein to new DNA synthesis ratio in heart and in muscle is much greater (∼4 fold) in Snell than in Rap, CR, or CL compared to their respective controls (Table S1) (Drake *et al*., 2014; Miller *et al*., 2014), which may suggest a potential relationship between an increase in proteostatic mechanisms and the degree of lifespan extension. The tissue and subfractional differences in synthesis rates between models may further suggest that the mechanism(s) for increased proteostasis between models is not universally shared but rather distinct for a given subcellular protein pool, tissue, and/or model (Table S1) (Miller *et al*., 2012a,b, 2014; Drake *et al*., 2013, 2014). However, the finding of an increase in the new protein to new DNA synthesis ratio in Snell dwarf mice in this study, combined with the same finding in other models of slowed aging, suggests that an increase in proteostatic mechanisms is a shared characteristic of long-lived models and thus represents an important target for the development of strategies to slow the aging process.

### mTORC1-related signaling

The mTORC1 signaling pathway is thought to be a key regulator of proteostasis due to its control over protein turnover and cell cycling (Laplante & Sabatini, 2012). Furthermore, decreased mTORC1 is a common characteristic of multiple long-lived models (Hsieh & Papaconstantinou, 2004; Harrison *et al*., 2009; Selman *et al*., 2009; Miller *et al*., 2012b; Drake *et al*., 2013). We found that rpS6 phosphorylation was decreased in the heart and liver from Snell mice compared to Con but did not find a statistically significant difference between groups in skeletal muscle, which may be reflective of the high variance in skeletal muscle rpS6. Also, 4E-BP1 phosphorylation was not different between Snell and Con in any tissue. It is important to note that the same statistical trends were maintained when sexes were combined and only the difference between Snell and Con assessed (Fig. S1). The lack of difference in the new protein to new DNA ratio between Snell and Con in the liver is in contrast to the decrease in mTORC1-dependent rpS6 phosphorylation in the Snell. Alternatively, skeletal muscle displayed an increase in proteostatic mechanisms without statistically significant changes in mTORC1-substrate phosphorylation, which, in the case of rpS6, has been noted in skeletal muscle of the similar Ames dwarf mouse (Sharp & Bartke, 2005). In the heart, the increase in the new protein to new DNA ratio in Snell mice corresponds with decreased rpS6 phosphorylation. We have shown increases in proteostatic mechanisms alongside increased mTORC1-substrate phosphorylation in the same tissues from age-matched long-lived CL mice (Drake *et al*., 2014). Differing outcomes in mTORC1-substrate phosphorylation in Snell skeletal muscle and liver further suggest that the increase in proteostatic mechanisms in slowed aging is not necessarily dependent on decreased mTORC1. The lack of consistency between inhibition of mTORC1 substrates related to protein synthesis and the increase in proteostatic mechanisms between models of slowed aging may suggest that other processes, such as autophagy and/or protein folding efficiency (Wang & Miller, 2012; Conn & Qian, 2013), which are partially regulated by mTORC1, are more pertinent to the increase in proteostatic mechanisms we have demonstrated in slowed aging models.

### Proteostasis and sex in Snell dwarf mice

Mean lifespan in female Snell with a DW/J background is increased by approximately 50% compared to normal controls, while male Snell dwarfs have an approximate 29% increase in mean lifespan compared to their respective sex-specific controls (Flurkey *et al*., 2002). With the exception of protein synthesis in the Mix fraction of skeletal muscle, there were no sex differences in protein or DNA synthesis, or mTORC1-substrate phosphorylation. Although differences in proteostatic mechanisms do not explain subtle sex differences in lifespan extension, it is important to note that both sexes have increased proteostatic mechanisms as well as significant lifespan extension.

### Summary and conclusions

By comparing new protein synthesis to new DNA synthesis, we illustrate that proteostatic mechanisms are uniformly increased across subcellular fractions in Snell skeletal muscle and heart even though rates of protein synthesis were variable when viewed without consideration of DNA synthesis. Additionally, we provide evidence that suggests that changes in proteostatic mechanisms are not necessarily dependent on changes in phosphorylation of protein synthesis-related mTORC1-substrates, which may suggest the importance of alternative processes (e.g., autophagy) in slowed aging. Together, with our previously examined long-lived models, we suggest that the maintenance of proteostasis is a shared characteristic of slowed aging.

## Experimental procedures

### Animals and treatments

All procedures and conditions at the animal care facility meet or exceed the standards for animal housing as described in the Animal Welfare Act regulations and the Guide for the Care and Use of Laboratory Animals. The University Committee on Use and Care of Animals at the University of Michigan approved all procedures. Snell dwarf and control mice were produced by crossing C3H/HeJ-Pit^dwJ^/+ females with DW/J-Pit1^dw/=^ males producing (DW/J × C3H/HeJ)F_1_ mouse offspring (Flurkey *et al*., 2001). The homozygous dw/dw dwarf offspring were compared to littermate controls using both dw/+ and +/+mice as indistinguishable controls (Vergara *et al*., 2004; Vitvitsky *et al*., 2013). After weaning (21–23 days), mice were housed in single-sex groups of 3–4 mice per cage (Flurkey *et al*., 2001). Animals were kept on a 12-h light and dark cycle and fed ad libitum.

At 7 months of age [age-matched to our previous investigations in long-lived models (Drake *et al*., 2014, 2013; Miller *et al*., 2012a,b)], Snell dwarf and control mice (*n* = 5 per sex, *n* = 10 per group) received deuterium oxide (D_2_O) for two weeks. After the labeling period and following an overnight fast, mice were euthanized using a CO_2_ overdose according to the AVMA Guidelines on Euthanasia. Complete loss of pedal reflexes was confirmed before tissues were collected. The posterior aspect of the distal hind limbs (gastroc complex—gastrocnemius, soleus, and plantaris [i.e., mixed skeletal muscle]), heart, liver, bone marrow from the tibia, and plasma via blood from cardiac puncture were taken and immediately frozen in liquid nitrogen for later analysis.

We assessed protein synthesis in mitochondrial-enriched (Mito), cytosolic (Cyto—other cytosolic organelles with the exception of mitochondria and nuclei), and mixed [Mix; nuclei, plasma membranes, and contractile proteins (heart and skeletal muscles)] subcellular fractions from skeletal muscle, heart, and liver (Neese *et al*., 2002; Busch *et al*., 2006; Miller *et al*., 2012a,b; Drake *et al*., 2013, 2014). Also, we assessed DNA synthesis according to procedures previously described (Neese *et al*., 2002; Busch *et al*., 2006; Miller *et al*., 2012a,b; Drake *et al*., 2013, 2014). Animals received an intraperitoneal injection of 99% enriched D_2_O to enrich the body water pool (assumed 60% of body weight) to 5% (Neese *et al*., 2002; Miller *et al*., 2012b; Drake *et al*., 2014). Animals then received 8% D_2_O in their drinking water with *ad libitum* access for 2 weeks.

### Protein isolation

Tissues were fractionated according to our previously published procedures (Robinson *et al*., 2010; Miller *et al*., 2012a,b; Drake *et al*., 2013, 2014). Tissues (15–50 mg) were homogenized 1:10 in isolation buffer (100 mm KCl, 40 mm Tris HCl, 10 mm Tris Base, 5 mm MgCl_2_, 1 mm EDTA, 1 mm ATP, pH = 7.5) with phosphatase and protease inhibitors (HALT, Thermo Scientific, Rockford, IL, USA) using a bead homogenizer (Next Advance Inc., Averill Park, NY, USA). After homogenization, subcellular fractions were isolated via differential centrifugation as previously described (Miller *et al*., 2012a,b; Drake *et al*., 2013, 2014). Once fraction pellets were isolated and purified, 250 μL 1 m NaOH was added and pellets were incubated for 15 min at 50 °C and 900 *g*.

### DNA isolation

Approximately 100 ng μL^−1^ (heart, gastroc complex) and 500 ng μL^−1^ (liver) of total DNA was extracted from ∼20 mg tissue (QiAamp DNA mini kit Qiagen, Valencia, CA, USA). DNA from bone marrow was isolated by extracting ∼300 mg from the tibial bone marrow suspension and centrifuged for 10 min at 2000 g.

### Sample preparation and analysis via GC/MS

#### Protein

Protein was hydrolyzed by incubation for 24 h at 120 °C in 6 N HCl. The hydrolysates were ion-exchanged, dried under vacuum, and resuspended in 1 mL molecular biology grade H_2_O. 500 μL of suspended samples wasderivatized [500 μL acetonitrile, 50 μL 1 m K_2_HPO_4_ (pH = 11), and 20 μL of pentafluorobenzyl bromide (Pierce Scientific, Rockford, IL, USA)], sealed, and incubated at 100 °C for 1 h. Derivatives were extracted into ethyl acetate. The organic layer was removed and dried by N_2_ followed by vacuum centrifugation. Samples were reconstituted in 1 mL ethyl acetate and then analyzed.

The pentafluorobenzyl-*N*,*N*-di(pentafluorobenzyl) derivative of alanine was analyzed on an Agilent 7890A GC coupled to an Agilent 5975C MS as previously described (Robinson *et al*., 2011; Miller *et al*., 2012a,b; Drake *et al*., 2013, 2014). The newly synthesized fraction (*f*) of proteins was calculated from the true precursor enrichment (*p*) using plasma analyzed for D_2_O enrichment and adjusted using mass isotopomer distribution analysis (MIDA) (Busch *et al*., 2006). Protein synthesis was calculated as the ratio of deuterium-labeled to unlabeled alanine (Busch *et al*., 2006) bound in proteins over the entire labeling period and expressed as fraction new in 2 weeks.

#### Body water

To determine body water enrichment, 125 μL of plasma was placed into the inner well of o-ring screw cap and inverted on heating block overnight. 2 μL of 10 m NaOH and 20 μL of acetone were added to all samples and to 20 μL 0–20% D_2_O standards and then capped immediately. Samples were vortexed at low speed and left at room temperature overnight. Extraction was performed by the addition of 200 μL hexane. The organic layer was transferred through anhydrous Na_2_SO_4_ into GC vials and analyzed via EI mode using a DB-17MS column.

#### DNA

Determination of ^2^H incorporation into purine deoxyribose (dR) of DNA was performed as previously described (Busch *et al*., 2006; Robinson *et al*., 2011; Miller *et al*., 2012b; Drake *et al*., 2013, 2014). Briefly, DNA isolated from whole tissue and bone marrow was hydrolyzed overnight at 37 °C with nuclease S1 and potato acid phosphatase. Hydrolysates were reacted with pentafluorobenzyl hydroxylamine and acetic acid and then acetylated with acetic anhydride and 1-methylimidazole. Dichloromethane extracts were dried, resuspended in ethyl acetate, and analyzed by GC/MS as previously described (Busch *et al*., 2006; Robinson *et al*., 2011; Miller *et al*., 2012b; Drake *et al*., 2013, 2014). The fraction new in 2 weeks was calculated by comparison with bone marrow (representing an essentially fully turned-over cell population and thus the precursor enrichment) in the same animal (Miller *et al*., 2012b; Drake *et al*., 2013).

### New protein to new DNA synthesis ratio

From the synthesis rates of protein and DNA, we calculated the protein synthesis to DNA synthesis ratio to be indicative of changes in proteostasis. This ratio illustrates how much new protein is made in relation to the rate of cellular proliferation (new DNA) during the labeling period (Drake *et al*., 2014; Miller *et al*., 2014).

### Western blotting

Western blots were completed on a portion of the Cyto fraction. Protein concentration was determined using a bicinchoninic acid assay (Thermo Fisher, Rockford, IL, USA). Samples were diluted to equal concentrations and boiled with Laemmli buffer, and then, 30–60 μg of protein was separated using 10% SDS-PAGE at 100 V. Proteins were transferred at 4 °C (100 V for 75 min in 20% w/v methanol, 0.02% w/v SDS, 25 mm Tris Base, 192 mm glycine, pH = 8.3) to nitrocellulose paper and incubated in 5% nonfat dry milk in Tris-buffered saline with Tween20 (TBST) for 1 h. Antibodies were purchased from Cell Signaling Technologies (Boston, MA, USA; rpS6 phospho-Ser[235/236] #4858, rpS6 total #2217, 4E-BP1 phospho [Thr37/46] #9459, 4E-BP1 total #9452) or Santa Cruz Biotechnology (Santa Cruz, CA, USA; -tubulin #sc-5274). Blots were incubated overnight with primary antibodies diluted 1:500 (skeletal muscle rpS6), 1:1000, 1:2000 (Liver rpS6), or 1:500 (β-tubulin). Blots were washed in 1x TBST and incubated with anti-rabbit or anti-mouse (β-tubulin) HRP-conjugated secondary antibody diluted 1:5000 in 5% milk with subsequent chemiluminescence detection (West Dura; Pierce, Rockford, IL, USA). Images were captured and densitometry-analyzed by UVP Bioimaging System (Upland, CA, USA). Blots were probed for phosphorylated proteins first, placed in stripping buffer (GM Biosciences, Rockville, MD, USA), and then re-probed for total protein. Equal loading was verified using ponceau-s staining and β-tubulin. Due to undetectable rpS6 in skeletal muscle and some heart samples, a portion of the homogenate was acetone-precipitated and then analyzed via western blotting.

### Statistics

Statistical analysis was performed using prism V4.0c (GraphPad Software, Inc. La Jolla, CA, USA). Treatment (Snell vs. Con) and subcellular fraction protein synthesis data and all sex comparisons were assessed by two-way anova. Combined sexes DNA synthesis data and treatment effect of individual subcellular fractions in heart were assessed via two-sided Student's *t*-test. Significance was set at *P* < 0.05, and *P*-values of ≤ 0.10 are noted. Data are presented as means ± standard error of the mean (SEM).

## Supporting Information

**Table S1** Summary of tissue and subfractional differences between multiple models of slowed aging. Significant differences between model and the respective control are indicated by directional arrows **Fig. S1** Western blotting of mTORC1 substrates rpS6 and 4E-BP1 in skeletal muscle, heart, and liver of Snell and Con with sexes combined.
