# Supplementary material for: Long-lived Snell dwarf mice display increased proteostatic mechanisms that are not dependent on decreased mTORC1 activity
Source: Aging Cell. 2015 Feb 26;14(3):474–82. doi: 10.1111/acel.12329 (PMC4406676; doi:10.1111/acel.12329)
Supplement: Supplementary file 1 [file acel0014-0474-sd1.pdf]

Table S1

Model	Tissue	Fraction	Protein Synthesis	DNA Synthesis	New Pro:DNA Ratio	rpS6	4E-BP1
Caloric Restriction (Ref 15 & 16)	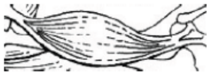	Mix	≈	≈	↑	↓	≈
		Cyto	≈		↑		
		Mito	↑		↑		
	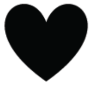	Mix	≈	↓	↑	↓	≈
		Cyto	≈		↑		
		Mito	≈		↑		
	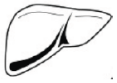	Mix	≈	↓	n/a	↓	≈
		Cyto	≈		n/a		
		Mito	≈		n/a		
Chronic Rapamycin Feeding (Ref 14)	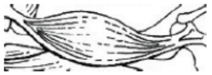	Mix	↓	↓	≈	↓	≈
		Cyto	↓		≈		
		Mito	≈		≈		
	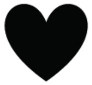	Mix	≈	↓	↑	↓	≈
		Cyto	≈		↑		
		Mito	≈		↑		
	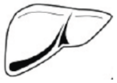	Mix	n/a	↓	n/a	↓	≈
		Cyto	n/a		n/a		
		Mito	n/a		n/a		
Crowded Litter (Ref 10)	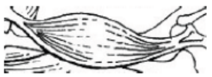	Mix	↑	≈	↑	≈	↑
		Cyto	↑		↑		
		Mito	↑		↑		
	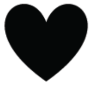	Mix	↑	↓	↑	↑	↑
		Cyto	↑		↑		
		Mito	↑		↑		
	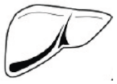	Mix	↑	≈	↑	↑	≈
		Cyto	↑		↑		
		Mito	↑		↑		
Snell Dwarf	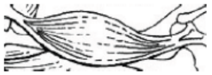	Mix	↓	↓	↑	≈	≈
		Cyto	↓		↑		
		Mito	↓		↑		
	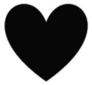	Mix	↓	↓	↑	↓	≈
		Cyto	↑		↑		
		Mito	↓		↑		
	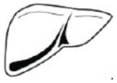	Mix	≈	≈	≈	↓	≈
		Cyto	≈		≈		
		Mito	≈		≈		

Figure S1

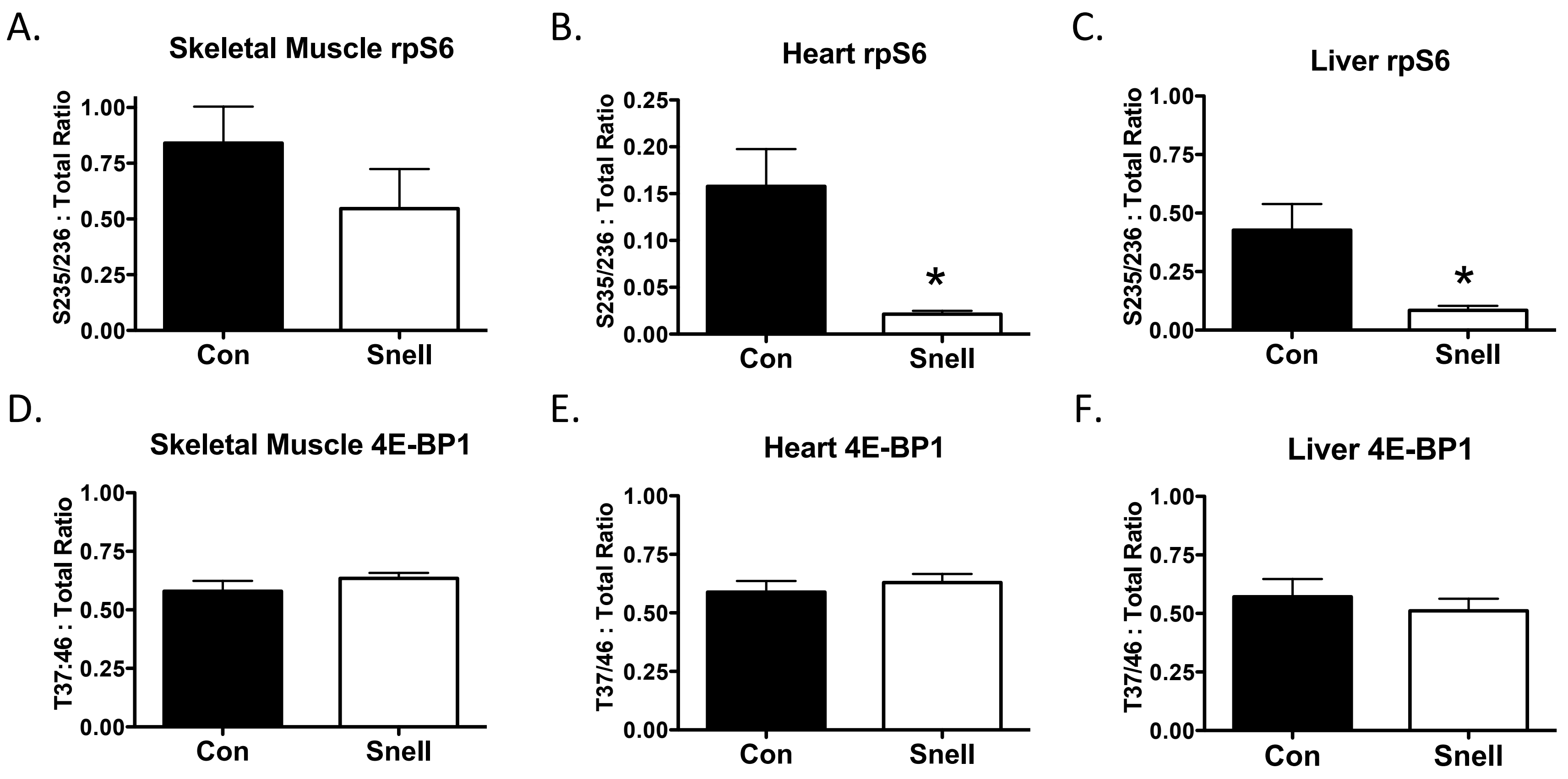
A.
Skeletal Muscle rpS6
S235/236 : Total Ratio
1.00
0.75
0.50
0.25
0.00
Con
Snell
B.
Heart rpS6
S235/236 : Total Ratio
0.25
0.20
0.15
0.10
0.05
0.00
*
Con
Snell
C.
Liver rpS6
S235/236 : Total Ratio
1.00
0.75
0.50
0.25
0.00
*
Con
Snell
D.
Skeletal Muscle 4E-BP1
T37:46 : Total Ratio
1.00
0.75
0.50
0.25
0.00
Con
Snell
E.
Heart 4E-BP1
T37/46 : Total Ratio
1.00
0.75
0.50
0.25
0.00
Con
Snell
F.
Liver 4E-BP1
T37/46 : Total Ratio
1.00
0.75
0.50
0.25
0.00
Con
Snell
